# Mucoadhesive PVA Film for Sustained Resveratrol Delivery: Formulation, Characterization, and Release Profile

**DOI:** 10.3390/molecules30122642

**Published:** 2025-06-18

**Authors:** Arleta Dołowacka-Jóźwiak, Izabela Nawrot-Hadzik, Adam Matkowski, Tomasz Ciecieląg, Agnieszka Gawin-Mikołajewicz, Ruth Dudek-Wicher, Mirosława Prochoń, Dorota Markowska, Robert Adamski, Adrian Wiater, Bożena Lucyna Karolewicz

**Affiliations:** 1 Department of Drug Form Technology, Wroclaw Medical University, Borowska 211 A, 50-556 Wroclaw, Poland; tomasz.ciecielag@student.umw.edu.pl (T.C.); agnieszka.gawin-mikolajewicz@umw.edu.pl (A.G.-M.); bozena.karolewicz@umw.edu.pl (B.L.K.); 2 Department of Pharmaceutical Biology and Biotechnology, Division of Pharmaceutical Biology and Botany, Wroclaw Medical University, 50-556 Wroclaw, Poland; izabela.nawrot-hadzik@umw.edu.pl (I.N.-H.); bbsekret@umw.edu.pl (A.M.); 3 Department of Pharmaceutical Microbiology and Parasitology, Faculty of Pharmacy, Wroclaw Medical University, 50-367 Wroclaw, Poland; ruth.dudek-wicher@umw.edu.pl; 4 Institute of Polymer and Dye Technology, Faculty of Chemistry, Lodz University of Technology, Stefanowskiego 16, 90-537 Lodz, Poland; miroslawa.prochon@p.lodz.pl; 5 Faculty of Process and Environmental Engineering, Lodz University of Technology, 90-924 Lodz, Poland; dorota.siuta@p.lodz.pl (D.M.); robert.adamski@p.lodz.pl (R.A.); 6 Department of Industrial and Environmental Microbiology, Institute of Biological Sciences, Faculty of Biology and Biotechnology, Maria Curie-Skłodowska University, Akademicka 19, 20-033 Lublin, Poland

**Keywords:** resveratrol, polyvinyl alcohol, *Reynoutria japonica*, physicochemical properties, oral mucosal, wound healing, polymeric film

## Abstract

This study aimed to develop and optimize polyvinyl alcohol (PVA)-based polymeric films containing resveratrol (RSV) and to evaluate their applicability as oral mucosal wound dressings. Given the dynamic and complex nature of the oral environment, physicochemical parameters such as elasticity, mucoadhesive strength, and the release profile of the RSV were systematically investigated. The therapeutic performance of pure resveratrol was compared with that of an extract derived from *Reynoutria japonica*. Films were fabricated using a solvent casting method and characterized in terms of thickness uniformity, weight, color consistency, and flexibility, all of which met the required pharmaceutical criteria. Two tested formulations, FR2 (RSV/PVA/PVP/MCA15C/NaCMC/W/PGE), FE2 (extract/PVA/PVP/MCA15C/NaCMC/W/PGE), showed the best mucoadhesive properties (261.11 ± 0.5 g for FR2 and 299.43 ± 0.38 g for FE2) and a favorable release profile both in water (72.42% for FR2, 77.23% for FE2) and in saliva (49.74% for FR2, 49.70% for FE2). Moreover, the optimized films are characterized by hydrophilicity (contact angle < 90°) and the pH value of the extract after their blurring is close to physiological, which promotes better tolerance and reduces the risk of irritation. Obtained results for polymeric films with resveratrol and *R. japonica* extract confirmed their great potential for use in dentistry as modern, mucoadhesive dressings, improving the effectiveness of local therapies.

## 1. Introduction

Resveratrol (RSV) is a plant-based polyphenol from the flavonoid group, whereby trans-resveratrol displays greater biological activity than the cis isomer. RSV is found mainly in the skin of red grapes, rhizomes and roots of *Polygonaceae*, and berry fruits as well as in wine [1,2,3,4]. Its compound with molecular weight is 228.25 Da with low water solubility (3 mg/100 mL), which limits its bioavailability. RSV is soluble in organic solvents such as ethanol and dimethyl sulfoxide (DMSO, 50 mg/mL). *Reynoutria japonica* Houtt. (syn. *Polygonum cuspidatum* Sieb. & Zucc., *Fallopia japonica* [Houtt.] Ronse Decr, common name: Japanese knotweed) of the mentioned *Polygonaceae* family, originating in East Asia, is currently considered an invasive species. The extract obtained from its rhizomes using ethanol, methanol, or water as solvents is the source of active pharmaceutical ingredients (APIs) such as resveratrol, rutin, catechins, and inulin. Due to its anti-inflammatory, antibacterial, and antioxidant properties, the extract is utilized in traditional medicine, the cosmetic industry, and dietary supplements. Recent research [4,5] has focused on its application in chronic diseases such as cardiovascular disorders, cancer, diabetes, and neurodegeneration, although most data derive from in vitro and animal studies. RSV is well absorbed in the small intestine, metabolized in the liver, and excreted in bile and urine, without individual metabolic differences [6]. It has strong antioxidant properties, neutralizing reactive oxygen species and chelating copper ions [7]. RSV has a strong anti-inflammatory effect by inhibiting cyclooxygenase enzymes (COX-1 and COX-2) and key inflammatory mediators such as NF-κB and GM-CSF. It has been shown in the study by Donnelly et al. [8] that RSV reduces the level of pro-inflammatory factors, such as IL-1β and IL-8, more effectively than quercetin. In addition, it supports tissue regeneration, including epithelium and bone tissue, and stimulates osteogenesis by activating the *SIRT1* and *RUNX2* genes. Incubation of bone marrow cells with RSV significantly increased the production of osteoblasts, especially at a concentration of 10–50 µm, indicating its potential in wound treatment and supporting bone reconstruction [9]. RSV has anti-aging effects by mimicking the effects of calorie restriction, which reduces oxidative stress and the production of ROS. In vivo studies in mice demonstrated that RSV improves vascular flexibility, motor coordination, and bone density and reduces endothelial damage [10]. RSV has an anti-proliferative effect, which is crucial to inhibit cancer development, where uncontrolled cell division leads to the formation of tumors. By inhibition of NF-κB activity and MIC-1 overexpression, RSV hinders the growth of tumors. The anti-proliferative effect of RSV has been demonstrated in pancreatic and liver cancers [11,12,13]. RSV also exhibits inhibitory activity against a wide range of pathogens, including viruses, bacteria, and fungi. Its antiviral effects are particularly pronounced at the early stages of viral replication, where it interferes with the expression of key viral proteins [14,15]. RSV displays a potent antifungal effect against dermatophytes, yeasts, and gray mold mainly by inducing apoptosis. It inhibits the development of fungi such as *Trichophyton* spp., *Microsporum* spp., *Saccharomyces cerevisiae*, and *Candida albicans*. In addition, RSV, although less effective towards bacterial species than fungi, inhibits the growth of Gram-negative and Gram-positive bacteria and bacterial biofilm, especially in the case of *Fusobacterium nucleatum*, which is enriched in lesions in periodontal diseases, halitosis, dental pulp infection, oral cancer, and systemic diseases [16,17,18].

Oral diseases include a wide range of conditions that can affect both soft and hard tissues, such as teeth. The most common is dental caries, caused by bacteria that convert sugars into acids, leading to enamel loss or deterioration. Factors such as diet, poor oral hygiene, and enamel erosion contribute to the development of this disease, which can result in pain, tooth loss, and speech problems [19]. Another widespread oral disease is gingivitis, caused by bacterial plaque, which leads to gum inflammation, bleeding, and pain. If left untreated, it can lead to tooth loss. The main objective of gingivitis treatment is to reduce the inflammation by removing dental plaque deposits and calculus. This can be managed if the patient starts following proper oral hygiene protocols or by professional procedures like scaling and root planning according to the severity of the condition [20]. Oral candidiasis, caused by *Candida* spp., is frequently observed in individuals with compromised immune systems and manifests as painful white or erythematous lesions. Various factors, including chronic diseases and antibiotic use, can promote its development. Effective prevention and management of oral diseases require adherence to proper oral hygiene practices and regular dental check-ups [21,22,23,24].

Modern research on natural pharmaceuticals, especially plant-based drugs, is gaining importance in dentistry, mainly due to antibiotic resistance, the cost of manufacturing synthetic drugs, and their potential adverse effects. Plant-based drugs can offer broad, beneficial effects, as well as diminish side effects and production costs. RSV is used in dentistry, especially in the treatment of caries, gingivitis, and periodontitis. Clinical studies have shown the effectiveness of a gel containing 2% RSV in reducing inflammation and gingival hyperplasia in orthodontic treatment. Resveratrol, applied in the form of spray, gel, tablets, and films, has also shown effectiveness in the treatment of gingivitis by improving saliva pH value, reducing dental plaque, and preventing tooth stains, especially in children. Studies on mucoadhesive tablets containing RSV or herbal formulations indicated the possibility of their use in the treatment of periodontitis. The results of studies [25,26,27,28,29,30,31,32,33,34] have proven that natural substances such as RSV can be an effective, safe, and economical solution in dentistry, both in the treatment and prevention of inflammation periodontal diseases, and can enhance the quality of orthodontic treatment.

Current research on RSV delivery systems in dentistry focuses on the development of formulations designed to enhance its bioavailability and therapeutic efficacy. Among these, polymeric films have emerged as promising carriers, offering prolonged retention at the application site and sustained, uniform release of the API. Mucoadhesive systems, including tablets and films, are particularly advantageous in pediatric dentistry, where the use of solid dosage forms can be challenging. Evaluations of these formulations include their dissolution, drug release profile, mucoadhesive properties assessments, and their effect on the mucous membrane. Tests on polymer films containing hydroxypropyl methylcellulose and ethylcellulose have demonstrated their good mechanical strength and long-term adhesion to the mucous membrane, which makes them promising carriers for RSV in dental therapy [35,36,37].

Based on the findings of our previous study [38], which demonstrated a complex release mechanism of bioactive compounds from *R. japonica* extract primarily RSV and piceid, the current research aims to optimize the formulation approach to further improve of their therapeutic performance. To this end, modifications in the film fabrication process were implemented to enhance extract bioavailability, ensure its uniform distribution within the polymer matrix, and enable modified release of resveratrol.

This study presents the development and in vitro evaluation of mucoadhesive polymeric films based on polyvinyl alcohol (PVA), incorporating RSV and *R. japonica* extract, intended for application on the oral mucosa. The formulation and manufacturing processes were optimized, and the resulting films were assessed with regard to critical quality attributes, including mechanical strength, mass and thickness uniformity, organoleptic characteristics, and mucoadhesive performance.

## 2. Results and Discussion

### 2.1. Visual Assessment

**Figure 1:**
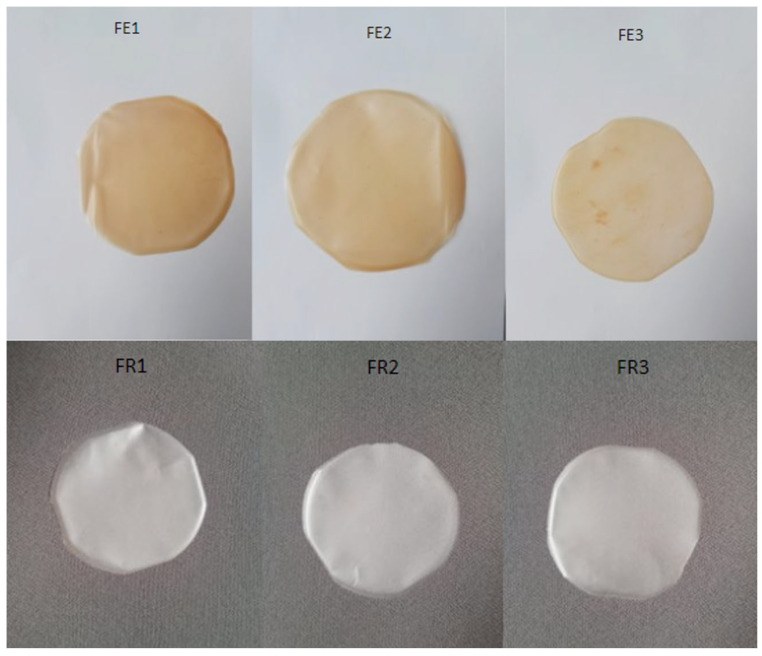
Images of formulation FE1 (extract/PVA/PVP/MCA400cP/NaCMC/W/PGE), FE2 (extract/PVA/PVP/NaCMC/MCA15C/W/PGE), FE3 (extract/PVA/PVP/MCA400cP/PGE), FR1 (RSV/PVA/PVP/MCA400cP/NaCMC/W/PGE), FR2 (RSV/PVA/PVP/NaCMC/MCA15C/W/PGE), FR3 (RSV/PVA/PVP/MCA400cP/PGE).

The tested formulations presented in Figure 1 were evaluated for key physical characteristics, including elasticity, presence of air bubbles, color, surface texture uniformity, and signs of delamination. Formulations FR1, FR2, FE1, FE2, and FE3 exhibited the most favorable properties, characterized by flexibility, smoothness, uniform color, and the absence of visible air bubbles or layer separation. Additionally, these samples were easily removed from the molds after drying and did not exhibit stickiness or residual viscosity, which indicates appropriate film-forming behavior. The formulations containing RSV (FR1–FR3) were milky-white, and those with the *R. japonica* extract were light brown (FE1–FE3). The FE3 formulation (extract/PVA/PVP/MCA400cP/PGE) showed non-uniform color and reduced elasticity compared to the other samples. Despite these imperfections, the formulations did not show visible air bubbles and could be easily removed from the molds. Furthermore, noticeable surface inhomogeneities were observed in the form of darker spots (Figure 1), probably resulting from local precipitation or aggregation of plant-derived compounds. These defects can be attributed to extract–polymer incompatibility at higher extract concentrations, insufficient mixing during formulation, or uneven drying conditions, leading to local changes in the structure and appearance of the films.

The formulations were also evaluated for uniformity of thickness, weight, and mucoadhesive strength. The results are presented in Table 1. The FR1, FR2, FE1, and FE2 formulations, which differ only in the source of resveratrol, were of similar thickness. The thickness of these formulations was less than the thickness of the FR3 and FE3 formulations. This is due to the fact that formulations FR1, FR2, FE1, and FE2 contained water that had evaporated during drying. The highest film thickness was observed in the case of the FE3 formulation. The thickness measurement obtained for the different fragment of the same film did not differ by more than 0.01 mm, indicating its uniformity and the correct selection of drying process conditions. FR2 formulation had the lowest average mass, while FE1 and FE2 had the same, and the highest average mass was FE3, which also had the highest thickness. The highest values of mucoadhesiveness were observed for formulations FR2 and FE2 (Table 1), which contained in their composition methylcellulose MCA 15C. This derivative was characterized by a higher viscosity than other semi-synthetic cellulose derivatives chosen in projected formulations. At the same time, its addition contributed to the greatest strength of mucoadhesion in optimized polymer film formulations.

The FR3 and FE3 formulations displayed the lowest mucoadhesive strength in vitro (Table 1). In these formulations, the quantity of methylcellulose derivatives used in the production process was the lowest, resulting in low mucoadhesion strength values. Formulations containing methylcellulose with a viscosity of 1200–1800 mPa·s (FE2: 299.43 g) obtained the highest mucoadhesion strength value. This suggests that both the concentration and the viscosity grade of methylcellulose significantly influence mucoadhesive performance. Higher viscosity polymers are known to form more robust gel networks upon hydration, leading to improved adhesion to mucosal surfaces. Moreover, the increased polymer content likely enhances the interaction with mucin, further contributing to stronger mucoadhesion. These findings are consistent with previous studies [37,38] highlighting the critical role of polymer characteristics in determining mucoadhesive behavior.

All tested formulations showed sufficient mechanical strength and obtained a positive result in the folding endurance test. None of the tested films were torn or cracked despite reaching 300 folds at an angle of 90°. Although the FE3 formulation showed less flexibility than the other formulations, it demonstrated sufficient resistance to bending. The result of this test confirmed the mechanical strength of all tested films.

### 2.2. FT-IR Spectroscopy Analyses

**Figure 2:**
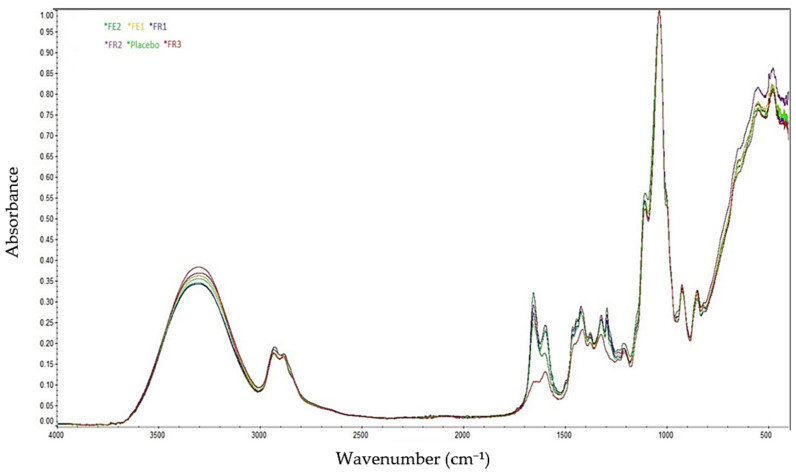
FTIR spectra of FE1 (extract/PVA/PVP/MCA400cP/NaCMC/W/PGE), FE2 (extract/PVA/PVP/NaCMC/MCA15C/W/PGE), FR1 (RSV/PVA/PVP/MCA400cP/NaCMC/W/PGE), FR2 (RSV/PVA/PVP/NaCMC/MCA15C/W/PGE), FR3 (RSV/PVA/PVP/MCA400cP/PGE), and placebo.

Figure 2 presents the FT-IR spectra of the tested formulations FE1, FE2, FR1, FR2, FR3, and the placebo. The characteristic absorption bands observed in the range of 3500–3000 cm^−1^ correspond to the stretching vibrations of CH groups and hydroxyl (OH) groups, which are typical for PVA and are present in all formulations.

The peaks between 1700 and 1500 cm^−1^ are attributed to C=O stretching vibrations (amide and carbonyl groups) and C=N or C=C stretching present in PVP. In the case of the FR3 formulation, where PVP was not added, the spectrum is less intense and different from other formulations. However, its presence may be due to interactions with other particles. In addition, a bond at 1100–1000 cm^−1^ is visible and corresponds to C–O–C and C–O stretching vibrations registered for methylcellulose derivatives such as NaCMC, MCA 15C, and MC 400 CP. Due to the low amount of RSV or *R. japonica* extract containing RSV, the bands specific to these substances (in the range of 1620–1500 cm^−1^ for RSV, corresponding to aromatic C=C and C=O groups) are not visible, and differences between the placebo and API formulations have not been identified. The FT-IR analysis confirmed the presence of key functional groups in the tested formulations. Characteristic broad bands observed between 3500 and 3000 cm^−1^ were attributed to O–H and C–H stretching vibrations, confirming the presence of polyvinyl alcohol across all samples. In formulations containing polyvinylpyrrolidone (PVP), additional peaks in the 1700–1500 cm^−1^ range corresponding to carbonyl (C=O), C=N, and aromatic C=C stretching were observed. These intermolecular interactions suggest enhanced polymer network formation and improved physicochemical stability. The FR3 formulation, which lacked PVP, exhibited reduced peak intensity, further supporting the role of PVP in polymer matrix structuring.

### 2.3. Contact Angle Measurement (Wettability)

As presented in Table 2, formulations FR2 and FE2 exhibited the highest contact angles compared to the other tested samples. This observation can be attributed to the presence of the less hydrophilic methylcellulose derivative MCA 15A used in these formulations. The differences in the average contact angle values between FR1, FE1 and FR3, FE3 were minor and can be considered negligible. These results provide an initial assessment of the formulations’ behavior in a physiological environment, particularly with respect to saliva wetting of the film surface.

A higher contact angle may indicate slower initial wetting, which could benefit prolonged residence time on the mucosa. Conversely, lower contact angles observed in other formulations suggest faster hydration, which may support quicker release of the resveratrol. These variations highlight the importance of carefully selecting polymer composition to tailor the balance between adhesion, hydration rate, and release kinetics.

The obtained contact angle values indicate the hydrophilic nature of the films, which promotes their wettability and influence on API release. Moreover, comparing wettability and release profiles suggests a correlation between increased hydrophilicity (lower contact angle) and immediate release of the RSV. Formulations with higher contact angles (FR2, FE2) exhibited release after lag time, likely due to slower hydration and substance diffusion of the polymer matrix. This supports the hypothesis that wettability plays a key role in modulating drug release kinetics and should be considered in designing mucoadhesive dosage forms.

### 2.4. Visualization of Polymer Crystalline Structure Using Polarized Optical Microscopy

**Figure 3:**
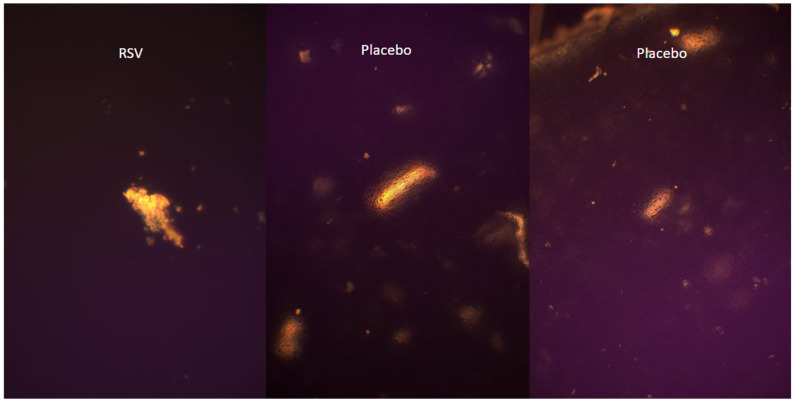
Resveratrol and placebo microscope image (RSV—pure RSV).

**Figure 4:**
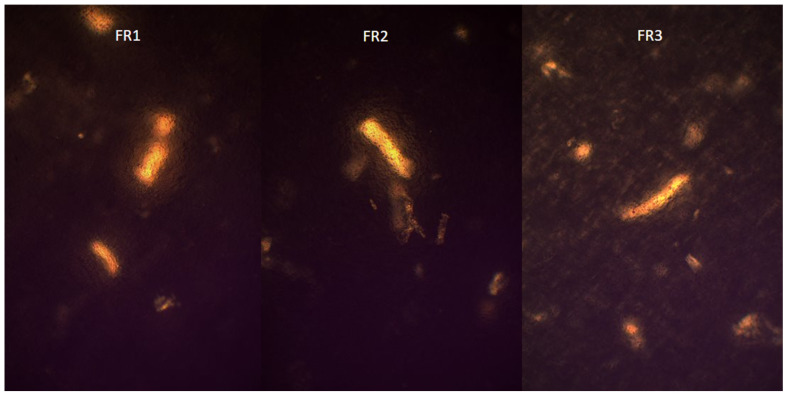
Microscopic image of formulations FR1 (RSV/PVA/PVP/MCA400cP/NaCMC/W/PGE), FR2 (RSV/PVA/PVP/NaCMC/MCA15C/W/PGE), FR3 (RSV/PVA/PVP/MCA400cP/PGE).

Microscopic analysis of the tested formulations (FR1, FR2, FR3) in comparison with placebo film and pure RSV did not show any evidence of crystallization of the resveratrol; microscopic images are presented in Figure 3 and Figure 4. The observed formations, present across all samples, are likely attributable to residual, partially undissolved polymers utilized during formulation preparation. This interpretation is supported by the fact that identical structures were observed both in the placebo film and in formulations containing RSV. Moreover, none of the samples displayed the characteristic crystalline structures clearly visible in the microscopic images of pure RSV. The lack of clear features of RSV crystallinity may indicate its amorphous dispersion in the polymer matrix or the possible formation of a molecularly dispersed system. Such a physical state is advantageous from a pharmaceutical perspective, as it may enhance the bioavailability of the API. In addition, the presence of RSV in an amorphous state may indicate its effective incorporation into the matrix without the risk of precipitation in the form of crystals, which is essential for the stability and homogeneity of the formulation. In the case of formulations containing *R. japonica* extract (FE1–FE3), no structures resembling the crystalline form of pure RSV were also observed.

### 2.5. In Vitro Resveratrol Release Profile

**Figure 5:**
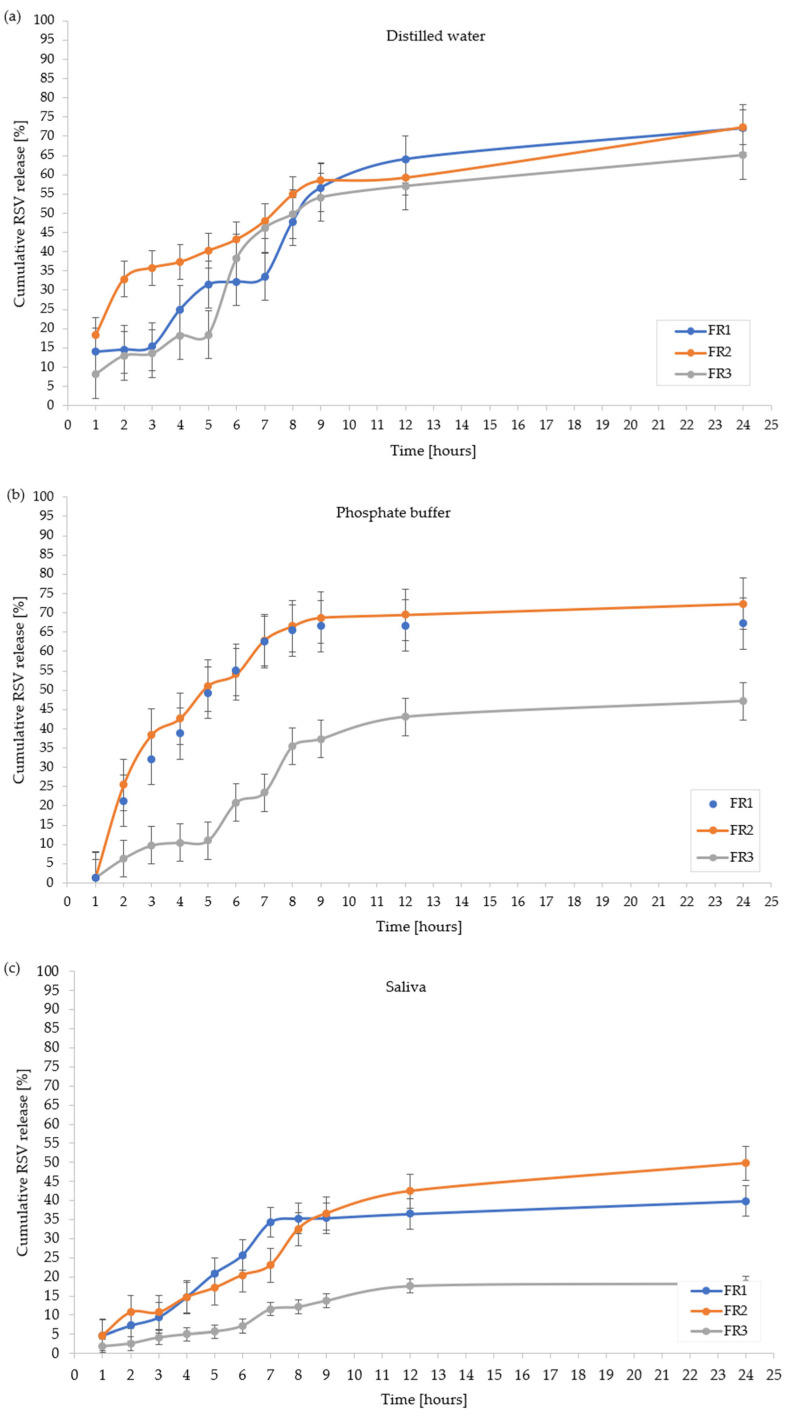

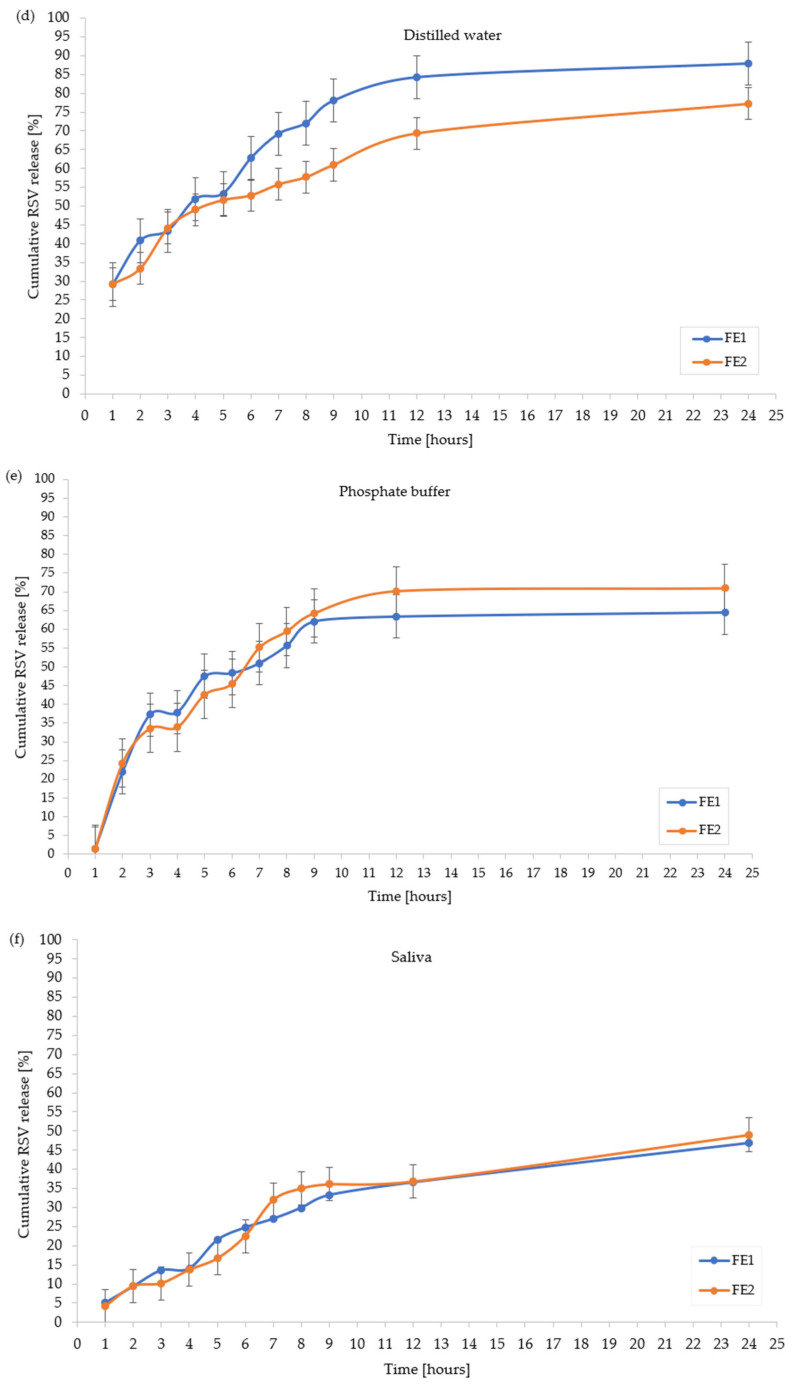
Cumulative release profiles of RSV from formulations FR1, FR2, and FR3 in (**a**) distilled water, (**b**) phosphate buffer, and (**c**) artificial saliva; and from formulations FE1 and FE2 with *R. japonica* extract in (**d**) distilled water, (**e**) phosphate buffer, and (**f**) artificial saliva. Data are presented as mean ± SD (*n* = 3). No statistically significant differences were observed between the formulations in any of the tested media (*p*  >  0.05).

The RSV release profiles from the developed formulations (FR1, FR2, FR3, FE1, and FE2) in three different media, distilled water, phosphate buffer, and artificial saliva, are presented in Figure 5 and in Table 3. Due to visible surface inhomogeneities and non-uniform coloration, the FE3 formulation was excluded from further analyses. The observed defects most likely result from incompatibility between the plant extract and the polymer matrix at higher extract concentrations, which may negatively affect the drug release profile and the reproducibility of the formulation’s physicochemical properties, and this requires further research.

The analysis of the RSV release profiles revealed significant differences, clearly demonstrating the influence of both the formulation composition and the dissolution medium on the RSV release from film. Regardless of the formulation tested, the lowest cumulative percent of RSV released was consistently observed in artificial saliva, likely due to its specific composition, which limits its capacity to facilitate RSV solubilization and diffusion. This effect may be attributed to the presence of mucins, enzymes, and inorganic ions such as calcium and phosphate, which can interact with the drug substance or the polymeric matrix, potentially forming complexes or creating a diffusion barrier. In contrast, distilled water and phosphate buffer provided more favorable conditions for RSV release.

As presented in Figure 5a, among the FR formulations tested in distilled water, FR2 and FR1 showed the most efficient release profiles, reaching approximately 72.42% and 72.06% of RSV released after 24 h, respectively, while FR3 showed a significantly lower RSV release in this time of 65.10%, respectively. FR2 demonstrated a more gradual and consistent release pattern, whereas FR1 exhibited a biphasic profile with an evident slowdown between the 5th and 7th hour, possibly reflecting matrix swelling followed by its erosion. FR3, lacking PVP, displayed a delayed and overall limited release profile. In phosphate buffer (Figure 5b), both FR1 and FR2 achieved similarly high release levels (approximately 73.15% and 84.12%, respectively), while FR3 differed significantly, reaching a maximum RSV release of 57.71%. In this medium, FR2 maintained a steady release throughout the test period, whereas FR1 showed an early rapid release phase up to the 6th hour, followed by a plateau. FR3 displayed a lag phase in release profile with slow, incomplete release over 24 h. In artificial saliva (Figure 5c), all formulations exhibited markedly lower RSV cumulative release, with FR2 performing best, reaching 49.70%, while FR1 and FR3 reached 39.86% and 18.28%, respectively, after 24 h. FR2 showed a continuous release profile without clear plateaus, while FR1 displayed a slowdown after the 7th hour. FR3 released RSV at a minimal rate, remaining far below the other formulations. For the FE formulations, Figure 5d illustrates that in water, FE1 exhibited superior RSV release, achieving 87.84% after 24 h, while FE2 reached 77.23%. Both formulations showed an initial rapid release phase during the first 5 h, although FE1 maintained a more sustained and higher release rate over the entire study period. In phosphate buffer (Figure 5e), FE2 provided the most favorable cumulative release (78.70%), while FE1 reached 66.70% after 24 h. Notably, FE2 demonstrated a nearly linear release profile, while FE1 exhibited a slight deceleration phase between the 3rd and 9th hour, indicating a possible matrix restructuring phase. In artificial saliva (Figure 5f), both formulations displayed limited RSV release, with FE2 slightly outperforming FE1 (48.80% vs. 49.70% after 24 h). Among the tested formulations, those containing PVP and MCA 15C (FR2, FE1, FE2) exhibited the most favorable release profiles in all tested media. This suggests that both PVP and MCA 15C play a critical role in enhancing RSV release, possibly by promoting matrix hydration, improving wettability, and facilitating diffusion of the RSV. Formulation FR3, which lacks PVP, was characterized by the lowest RSV release across all conditions, further confirming the essential function of PVP and MCA 15C in modulating the release profile.

### 2.6. Disintegration Test

Table 4 summarizes the pH values of extracts after film disintegration and the observed disintegration behavior of each optimized polymer formulation.

These results emphasize the crucial role of the surrounding medium in determining the structural integrity and disintegration behavior of the obtained formulations. Aqueous environments, especially artificial saliva, clearly promote their disintegration as a result of hydration of the film matrix, mimicking conditions in the oral cavity. This is particularly important for oromucosal formulations, where rapid disintegration is desired for effective drug release and absorption across the mucosal membrane. The FE2 and FE3 formulations demonstrated complete disintegration in distilled water, suggesting their high susceptibility to moisture, an attribute potentially beneficial for quick therapeutic action. In contrast, films exposed to DMSO remained intact in all tested formulations, underscoring the solvent’s strong capacity to preserve the film structure. This phenomenon is likely attributed to DMSO’s aprotic, non-aqueous nature, its high polarity, and basic pH, which collectively inhibit polymer swelling and erosion by preventing the hydration processes critical for film disintegration. Such properties can be advantageously employed in pharmaceutical technology during processing, handling, or storage, where maintaining the structural integrity of the film is desirable. The FE3 formulation was the only one that remained flat and transparent in DMSO, which could indicate better polymer miscibility or homogeneity in this specific film. The similar pH values recorded post-disintegration across formulations and media suggest that no significant interactions or degradation products were released that could alter the medium’s pH. This indicates the chemical stability of the film components upon contact with physiological and non-physiological environments. The observed correlation between disintegration behavior and media type provides valuable insights into the potential in vivo performance of the tested films and underscores the importance of media selection in formulation testing protocols.

### 2.7. Summary of Results

The collected data clearly indicate that the prepared formulations (with the exception of FE3) meet the quality requirements for optimization and exhibit the desired properties (Table 1, Table 2, Table 3 and Table 4). In terms of visual characteristics, most formulations showed a uniform appearance, consistent color, and no visible air bubbles. The removal of the films from the molds after drying was smooth, and their thickness remained consistent, which was further confirmed by mass measurements. The FE3 formulation, however, deviated from the others in terms of appearance, thickness, and weight, highlighting the need for further optimization. All tested films demonstrated good mechanical resistance and high mucoadhesive strength. Contact angle measurements confirmed the hydrophilic nature of all films, with values below 90°. The lowest wettability was observed for FR2 and FE2, which may contribute to their greater stability in aqueous environments. The RSV release profiles met the predefined quality criteria. The FE1 formulation achieved the highest release of RSV in distilled water (87.84%), while FR2 showed the highest release in phosphate buffer (84.12%) and artificial saliva (49.74%). Notably, artificial saliva yielded the lowest RSV release values across all formulations, possibly due to interactions with its components. These findings suggest that artificial saliva may form a diffusion-limiting barrier and should be prioritized in further release studies to better simulate in vivo conditions. All extracts obtained after film disintegration in the tested media had near-neutral pH values. The highest pH was observed for FE2 (7.43) disintegrated in water, and for FR3 (7.65) in artificial saliva solution. The lowest pH values were recorded after disintegration of FR3 in water (6.80) and FR2 in artificial saliva (7.36), yet the variations were minimal and most likely reflect the buffering capacities of the respective media rather than any degradation of the films. This supports the chemical stability and biocompatibility of the formulations. The Franz diffusion cell method proved unreliable for this type of thin polymer film, likely due to membrane interference or the mechanical fragility of the films. As such, future studies should consider alternative release testing methods, such as modified dissolution systems or in vitro mucosal models. The distinctive characteristics of the FE3 formulation—its inconsistency in appearance, thickness, and mass, as well as the absence of RSV release data—may suggest inhomogeneous polymer distribution or incomplete film formation during processing. Despite meeting mechanical and mucoadhesive criteria, these observations underline the need to revisit and refine its composition or manufacturing parameters. Interestingly, FE2 exhibited the highest mucoadhesive strength and the highest contact angle, indicating that stronger adhesion does not necessarily correlate with higher wettability. This underscores the complex interplay between formulation composition and functional performance, where even slight variations in polymer content or type can markedly influence film behavior. FE1 emerged as the most effective formulation in water, consistent with its lower contact angle, suggesting enhanced hydration and diffusion capacity. In contrast, FR2 demonstrated superior release in phosphate buffer and artificial saliva, positioning it as a promising candidate for oromucosal applications where rapid drug release under physiological conditions is desired.

## 3. Materials and Methods

### 3.1. Materials, Reagents, and Apparatus

#### 3.1.1. Materials and Regents

This study utilized the following reagents: polyvinyl alcohol (PVA, Mwt 85,000–124,000, degree of hydrolysis 98–99%, CAS: 9005-89-5; Sigma-Aldrich, MO, USA); polyvinylpyrrolidone (PVP, K90, Mwt 40,000, CAS: 9002-89-5; Sigma-Aldrich, St. Louis, MO, USA); methylcellulose (MC 400 cP, CAS: 9004-67-5; Sigma-Aldrich, MO, USA); methylcellulose (MCA 15C, viscosity 1200–1800 mPas, CAS: 9004-67-5; Sigma-Aldrich, MO, USA); sorbitol (CAS: 50-70-4; Sigma-Aldrich, MO, USA); sodium carboxymethylcellulose (NaCMC, viscosity 300–600 mPas, CAS: 9004-32-4; VWR Chemicals, Solon, Ohio, USA); propylene glycol (PGE, 85%, CAS: 56-81-5; Fagron, Modlniczka, Poland); ethanol (99.8%, CAS: 64-12-5; Chempur, Piekary Śląskie, Poland); methanol (pure, analytical grade, CAS: 67-56-1; Chempur, Piekary Śląskie, Poland); sodium dihydrogen phosphate (NaH_2_PO_4_, CAS: 7558-79-4; Pharma Cosmetic, Kraków, Poland); sodium chloride (NaCl, CAS: 7647-14-5; Pharma Cosmetic, Kraków, Poland); calcium chloride dihydrate (CaCl_2_·2H_2_O, CAS: 10035-04-8; Biochem, Warsaw, Poland); magnesium chloride (MgCl_2_, CAS: 7786-30-3; Biochem, Warsaw, Poland); potassium chloride (KCl, CAS: 7447-40-7; Chempur, Piekary Śląskie, Poland); acetonitrile (CAS: 75-05-8; Chempur, Piekary Śląskie, Poland); phosphate buffer concentrate pH 6.8 (PBS, CAS: 7559-79-4; Alchem, Toruń, Poland); propylene glycol (PG, CAS: 57-55-6; Pure Chemical, Warsaw, Poland); dimethyl sulfoxide (DMSO, CAS: 67-68-5; Chempur, Piekary Śląskie, Poland); purified LC-MS-grade water (CAS: 7732-18-5; J.T. Baker, Phillipsburg, NJ, USA).

Materials used in this study included polystyrene Petri dishes with a surface area of 78.5 cm^2^ (Imanex), non-sterile zip-lock bags (LDPE, BRB), sterile zip-lock bags (Chemivet), pipette tips for automatic pipettes: 200 µL, 500 µL, 1000 µL, and 5000 µL (Labmate Pro, PZ HTL, Warsaw, Poland), Parafilm (Bemis Inc., Neenah, WI, USA), PTFE syringe filters with a pore size of 0.22 µm (Merck Millipore Ltd., Burlington, MA, USA), Mini-UniPrep G2 filtration vials (Cytiva, Marlborough, MA, USA), injection needles 0.8 mm × 16 mm (KDM), Spectra/Por dialysis membrane discs made of regenerated cellulose with a molecular weight cut-off (MWCO) of 12,000–14,000.

#### 3.1.2. Apparatus

Apparatus and equipment included the following: a laboratory drying oven (Pol-Eko-Aparatura, SLN 115 Simple, Wodzisław Śląski, Poland); pH meter + electrode ERH-12-6 no. 2071 (CPC-511 EMETRON, Lublin, Poland); texture analyzer (TA.XT Plus, Stable Micro Systems, Godalming, UK); sterilizer MLS-3750 (SANYO Electric Co., Osaka, Japan); analytical balance PA2/02CM/1 (Ohaus Pioneer, Parsippany, NJ, USA); laboratory stirrers (Labinco LD846, Labinco BV, Breda, Netherlands); ultrasonic bath Al.-04-12 (Advantage-LAB, Gent, Belgium); shaking water bath (Memmert GmbH + Co. KG, Schwabach, Germany); hydraulic press with Specac 13 mm DIE PT 3000 mold (Specac, Orpington, UK); freezer (Gorenje, Velenje, Slovenia); automatic pipettes 5–50 µL, 100–1000 µL, and 1000–5000 µL (HTL, Labmate PRO, Warsaw, Poland); digital caliper (Hard Head, Skara, Sweden); HPLC system (Ultimate 3000 RS, Thermo Dionex, Sunnyvale, CA, USA), which was equipped with a low-pressure quaternary gradient pump, a vacuum degasser, an autosampler, a column compartment (Kinetex C18 2.6 µm, 150 mm × 2.1 mm, Phenomenex, Torrance, CA, USA), and DAD (Diode Array Detection); goniometer (Contact Angle Goniometer, Ossila Ltd., Sheffield, UK); laminar flow hood (Mars Safety Class 2, SCANLAF A/S, Lyskær, Denmark); laboratory centrifuge ROTINA 420 (Hettich Lab Technology, Tuttlingen, Germany).

#### 3.1.3. Preparation of *Reynoutria japonica* Extract

The study material consisted of mature, undamaged, fully developed (diameter 15–30 mm) rhizomes of *R. japonica* collected from urban areas near Wrocław. Species identification was made by specialists from the Medicinal Plant Botanical Garden.

To obtain the dry extract, 50 g of air-dried rhizomes were powdered at the Department of Biology and Pharmaceutical Botany of the Wrocław Medical University. Extraction was carried out for 2 h in an ultrasonic bath using 500 mL of 25% ethanol. The solvent was evaporated under reduced pressure to obtain a dry extract.

### 3.2. Preparation of a Water–Ethanol Solution with RSV

Exactly 10 mg of pure RSV was weighed and dissolved in 10 mL of 25% ethanol solution. This solution was then used as the stock solution for preparing the calibration curve described in the subsection “Calibration curve for RSV content”.

### 3.3. Preparation of Phosphate Buffer

A phosphate buffer solution with pH 6.8 was prepared from a concentrate, following a modified pharmacopoeial method based on the Polish Pharmacopoeia XIII [39]. The preparation involved dissolving 1 g of concentrate in 25 g of distilled water suitable for HPLC, in accordance with the manufacturer’s instructions on the packaging.

### 3.4. Preparation of Artificial Saliva Solution

To prepare the artificial saliva solution, the following reagents were weighed: NaCMC—4.400 g; NaCl—0.380 g; KCl—0.540 g; CaCl_2_ × 2 H_2_O—0.0065 g; MgCl_2_—0.020 g; sorbitol—13.50 g; and Na_2_HPO_4_—0.150 g. All ingredients were subsequently dissolved in 450 mL of HPLC-grade water and mixed until fully dissolved.

### 3.5. Solubility Assessment of RSV and Extract of Reynoutria japonica

**Figure 6:**
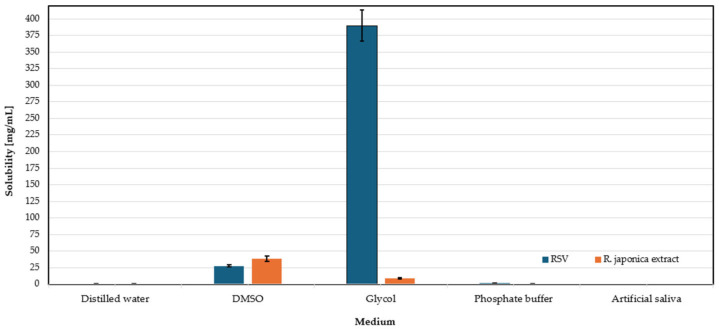
Solubility of RSV and *R. japonica* extract in tested media.

This study aimed to identify the most suitable solvent medium for dissolving RSV and extracts of *R*. *japonica* that would best reflect the physiological conditions of the oral cavity. Solvents were selected based on their low toxicity and minimal irritation risk, making them appropriate for dental applications. The tested solvents included distilled water, phosphate buffer (pH 6.8), propylene glycol, dimethyl sulfoxide, and artificial saliva. An excess amount of RSV and extract was added to Mini-UniPrep vials with PTFE filters (0.20 µm) containing 2 mL of each solvent. Samples were protected from light and incubated for 24 h at 37 °C using a laboratory shaker. After incubation, some samples were kept at room temperature and then centrifuged for 30 min at 3000 rpm. The resulting solutions were diluted and prepared for HPLC analysis. The solubility results of RSV and the extract are presented in the graphs shown in Figure 6.

In the case of the *R. japonica* extract, the highest solubility was observed in DMSO (38.52 mg/mL), followed by glycol (8.65 mg/mL), then phosphate buffer (0.068 mg/mL), and distilled water (0.053 mg/mL). In this case, the lack of data regarding solubility in artificial saliva is due to the same reason as in the case of RSV solubility testing. This study shows that RSV and the extract have various solubility properties and require the use of different optimal media, with glycol being optimal for RSV, while DMSO is optimal for the extract.

### 3.6. Methods

This study included the optimization of the technology polymer films formulated, an analysis of their properties, and optimization of the resveratrol release method. The first stage involved the solubility of RSV and *R. japonica* extract in various solvents. In the second stage, the technology for preparing placebo and polymer films with RSV or extract was optimized using cyclic freezing and thawing. The physicochemical properties of the polymers, such as structure, solubility, and viscosity, were evaluated in the context of obtaining mechanically durable dressings. The next stage involved the analysis of films in terms of their morphology, mechanical properties, adhesion, and the release profile of RSV.

#### 3.6.1. Polymer Film Preparation Technology

During the optimization of polymer film preparation, 6 formulations were prepared according to Table 5, and the detailed composition of the films is presented in Table 6, Table 7, Table 8, Table 9, Table 10 and Table 11. The optimized polymer films were obtained in a laminar chamber by casting from a solution, according to Table 12. In the first stage, aqueous solutions of the polymers were prepared at appropriate concentrations. The PVA solution was obtained by dissolving PVA in distilled water, mixing it for 24 h at 70 °C, and then sterilizing it at 121 °C for 20 min. The PVP solution was prepared by dissolving PVP in distilled water and mixing it for 24 h at room temperature, followed by sterilization at 121 °C for 20 min. Methylcellulose solutions, differing in viscosity (MC 400 cP, MCA 15C, NaCMC), were prepared by adding methylcellulose to water, leaving it for 24 h to swell, mixing it for 3 h, and sterilizing it at 121 °C for 20 min.

During the optimization of the placebo polymer film preparation process, a series of formulations were developed and subjected to stages of physical PVA crosslinking. This process involved cyclic freezing and thawing the polymer mixtures at temperatures ranging from −18 °C to +22 °C. Polymer crystallization occurred during the cyclic freezing and thawing, leading to increased film crosslinking. The freezing and thawing cycle was repeated at various time intervals, and after each freezing stage, the formulation solutions were mixed at room temperature to achieve a homogeneous polymer dispersion. The mixtures were then subjected to sonication in an ultrasonic bath to remove air bubbles for 15 or 30 min. The formulation solutions underwent five freezing and thawing cycles, during which the mixtures were frozen at −18 °C and then thawed at room temperature, adjusted according to the formulation composition.

The optimized polymer films were obtained by casting from a solution in a laminar chamber, as outlined in Table 5. Three types of formulations containing RSV and three types with *R. japonica* extract were prepared. The RSV formulations were labeled FR1, FR2, and FR3, while those with *R. japonica* extract were labeled FE1, FE2, and FE3. Appropriate amounts of PVA and PVP were mixed to prepare the optimized polymer films, followed by adding an ethanolic–aqueous solution containing *R. japonica* extract or RSV. Before adding the extract, it was micronized in a mortar. After this stage, PVA underwent preliminary crosslinking through freezing and thawing cycles. Methylcellulose solutions, glycerol, and water were then added to the formulations. The formulations were subjected to the proper crosslinking process, which was supported by sonication and mixing to ensure uniform crosslinking of the films.

The next step involved removing air bubbles from the solution, followed by additional sonication. The prepared formulations were cast onto petri dishes in 30 g portions, then subjected to vacuum and drying.

#### 3.6.2. Methods of the Assessment of Polymeric Films with Plant Material

1. Visual Assessment

The obtained formulations (FR1, FR2, FR3, FE1, FE2, FE3) were evaluated for flexibility, the presence or absence of air bubbles, color, uniformity, consistency, and potential separation. This assessment was performed for initial organoleptic evaluation with the naked eye, assessing the formulations for correctness, usability, and applicability to mucous membranes of the oral cavity and their potential for further research use.

2. Measurement of Mass and Thickness

The uniformity of mass was tested on three samples of the final dried formulation, with dimensions 2.5 cm × 2.5 cm, taken from three random areas. The measurements were conducted using an analytical balance with an accuracy of 0.1 g. The results in Table 1 are presented as the average of three measurements for each formulation and the calculated standard deviation. The thickness of the formulations was measured on three samples (2.5 cm × 2.5 cm), using an electronic caliper with an accuracy of 10 µm. Measurements were taken at the corners and the center of each sample, and the results are presented as the average of five measurements for three samples of each formulation, with the standard deviation.

3. Bending Resistance Test

The mechanical properties of the obtained polymer films were tested according to the method described by Vecchi et al. [36]. This study aimed to confirm the sufficient mechanical strength and flexibility of the final films. Three samples with dimensions 2.5 cm × 2.5 cm were taken from three randomly selected areas of formulations FR1, FR2, FR3, FE1, FE2, and FE3. These samples were folded in half at a 180° angle in the same place. This action was performed until the film broke or cracked. The results are presented as the average of the measurements with the standard deviation.

4. Mucoadhesion Strength Test

The mucoadhesion test was conducted on three 2.5 cm × 2.5 cm film samples for each formulation (FR1, FR2, FR3, FE1, FE2, FE3) using the TA.XT Plus texture analyzer (Stable Micro System, Godalming, UK). A movable arm attachment and the A/Muc mucoadhesion probe were used. The test involved 13 mm mucin disc made by compressing 250 mg of the substance attached to the movable arm. The disc was then immersed in a 5% mucin solution and placed in a beaker with artificial saliva, where the film sample was placed. The movable arm was lowered into the beaker at a speed of 1 mm/s, and the system was heated to 37 ± 1 °C. The force applied to the film was 0.1 N for 30 s. After this time, the arm was raised, and the program collected data on the force required to detach the mucin disc from the film. The results were presented as the average of three repetitions for each formulation.

5. FT-IR Spectroscopy

This study was conducted to observe molecular interactions between the components of the polymer film formulations and the RSV and the *R. japonica* plant extract. The Thermo Nicolet iS50 device (Waltham, MA, USA) with a Fourier transform was used in this analysis. An ATR attachment was used to observe the FTIR-ATR spectrum. Film samples with dimensions 2.5 cm × 2.5 cm were used for the study. The observation was conducted in the wavelength range of 400–4000 cm^−1^ with a scanning speed of 0.2 cm/s. The analysis was performed for all prepared polymer film formulations and the placebo.

6. Visualization of Polymer Crystalline Structure Using Polarized Optical Microscopy

The morphology of the polymer film formulations with RSV was examined using a microscope equipped with polarized light observation. The experiment aimed to exclude any potential crystallization of RSV in the formulations containing pure RSV. Observations were made of pure RSV, placebo polymer film, and all three formulations containing RSV. The study gradually increased the magnification, with each formulation being observed at a 100× magnification.

7. Contact Angle Measurement

The wettability angle was measured for all polymer films. The test was performed on 2.5 cm × 2.5 cm samples, and the contact angle was measured with an accuracy of 0.01° using a goniometer with a built-in digital camera. This experiment was based on the assumption that the droplet’s shape is a sphere segment, and knowing its height and volume allows for calculating the contact angle. The test was conducted by placing a single film sample on a stand, then applying a 50 µL drop of purified water using a micropipette. The contact angle, formed between the surface of the film and the tangent to the liquid surface, was measured using the camera and dedicated software. Measurements were taken for three fragments of film cut from different areas of the dried formulation. The results are presented as the average value of three measurements with the standard deviation to estimate the measurement precision. The study results are presented in Table 2 as an average of three measurements for each of the optimized polymer film formulations, with the standard deviation. A liquid is considered to “wet” the surface when the contact angle is less than 90°, and the surface is not “wetted” when the contact angle is greater than 90°.

8. Determining the Release Profile of Resveratrol from Film Formulation

To investigate the release profile of RSV from the film formulation, a study was conducted in a thermostated water bath at 37 ± 1 °C with horizontal shaking at 60 cycles per minute. Formulation FE3 was excluded from the test due to not meeting the organoleptic requirements. The study was conducted for the remaining formulations: FR1, FR2, FR3, FE2, and FE2 in three different media: water, phosphate buffer at pH 6.8, and artificial saliva solution. Film fragments measuring 2.5 cm × 2.5 cm were carefully weighed and placed in beakers containing 10 mL of the appropriate media. The beakers were tightly sealed with Parafilm and placed in the water bath, where 100 µL samples were taken at time intervals of 1, 2, 3, 4, 5, 6, 7, 8, 9, 12, and 24 h. After each sample collection, the medium volume was replenished to 10 mL, and the solution was centrifuged for 10 min at 5000 rpm. The samples were then filtered through PTFE filters with a pore size of 0.22 µm and analyzed by HPLC. The content of RSV and extract components was determined using the method described by Nawrot-Hadzik et al. [36], with an HPLC system equipped with a DAD detector and a Kinetex C18 column. A gradient elution was applied with formic acid solutions in water and acetonitrile. The entire analysis process was repeated three times, and the results were calculated based on a calibration curve, determining the amount of RSV in the released samples

9. Study of the RSV Content in A Single Carrier and pH of Extract Measurement after Film Disintegration

FR1, FR2, FR3, FE1, FE2, and FE3 formulations were fully dissolved in five different media: distilled water, phosphate buffer at pH 6.8, glycol, dimethyl sulfoxide, and artificial saliva solution. For this purpose, film fragments with dimensions 2.5 cm × 2.5 cm were accurately weighed and placed in beakers containing 10 mL of the appropriate medium. The beakers were tightly sealed with parafilm and placed in a water bath at 37 ± 1 °C, shaking at 60 cycles per minute. After 24 h, the degree of film disintegration was assessed, and the solutions containing the films were mixed for approximately 1 min to obtain a uniform sample for pH measurement. The pH measurement was performed to ensure no significant reactions occurred between the films, their components, and the medium used. pH measurements were made using a combined electrode in a pH meter at a temperature of 21 ± 1 °C, with an accuracy of 0.01 pH unit. The measurement was taken until the pH value stabilized, indicating the completion of the measurement.

For RSV content, a single carrier of 1 mL sample was taken from each beaker, and centrifuged for 10 min at 5000 rpm. After centrifugation, 900 µL of 80% (m/m) methanol was added to the sample, which was then filtered through PTFE filters with a pore size of 0.22 µm. The prepared solutions were subjected to qualitative and quantitative analysis using HPLC, following the method described by Nawrot-Hadzik et al. [37]. The content of RSV was calculated based on the calibration curve.

### 3.7. Statistical Analysis

All experiments were performed in at least triplicate, and the results are expressed as mean values ± standard deviations (SDs). Statistical significance between groups was evaluated using two-way analysis of variance (two-way ANOVA) with GraphPad Prism software (version 9.0; GraphPad Software, San Diego, CA, USA).

## 4. Conclusions

This study aimed to optimize the manufacturing technology of PVA polymer films containing RSV and *R. japonica* extract. The presented data from the experiments confirm that the research assumptions were met and optimized films were successfully obtained. The developed films exhibited desirable visual and mechanical properties, proving a stable and reproducible technological process. It has been shown that the studied films demonstrated appropriate elasticity, mucoadhesive strength, wettability, disintegration, and resveratrol release profiles under physiological conditions—parameters essential for dressings intended for use in the dynamic environment of the oral cavity. Importantly, pH measurements taken after film disintegration across different media (distilled water, buffer, artificial saliva) remained within a physiologically acceptable range of approximately 6.8 to 7.6. This pH range is considered safe and non-irritating for the oral mucosa, which typically maintains a pH around 6.2–7.6. Even after full film disintegration, the absence of significant pH deviations suggests chemical stability of the components. It confirms that the formulations are unlikely to disrupt the natural mucosal barrier or cause discomfort. Therefore, the optimized films can be considered biocompatible and safe for intraoral application. FR2 and FE2 emerged as the most promising candidates for further development among the tested formulations. They displayed the highest mucoadhesive strength and the most favorable RSV release profiles. Standard release tests confirmed the potential for sustained local substance delivery, particularly relevant in dental applications.

In conclusion, the optimized polymer films containing RSV and *R. japonica* extract show strong potential for dental applications due to their favorable physicochemical properties, mucosal safety, and the well-documented therapeutic potential of RSV.

## Figures and Tables

**Table 1:** Results of measurements (with standard deviation values) of the average thickness, mass, and mucoadhesion strength of tested formulations.

Formulation	FR1	FE1	FR2	FE2	FR3	FE3
Average thickness [mm]	0.14 ± 0.01	0.15 ± 0.01	0.15 ± 0.01	0.15 ± 0.01	0.18 ± 0.01	0.22 ± 0.02
Average mass [mg]	157 ± 3.0	138 ± 2.5	129 ± 1.8	138 ± 2.8	154 ± 4.0	159 ± 2.5
Average mucoadhesiveness [g]	197.22 ± 0.75	197.45 ± 0.5	261.11 ± 0.5	299.43 ± 0.38	175.78 ± 0.36	180.33 ± 0.6

**Table 2:** Results of the contact angle test.

Formulation	FR1	FE1	FR2	FE2	FR3	FE3
Average contact angle [°]	48.76 ± 0.01	48.87 ± 0.01	55.07 ± 0.02	58.28 ± 0.02	48.45 ± 0.01	49.02 ± 0.01

**Table 3:** Cumulative percentage RSV released from formulations (FR1–FR3, FE1–FE2) in various media after 24 h.

Test	RSV Release [%]				
	FR1	FR2	FR3	FE1	FE2
Distilled water	72.06 ± 2.21	72.42± 2.32	65.10 ± 1.75	87.84 ± 1.97	77.23 ± 2.11
Phosphate buffer	73.15 ± 2.99	84.12 ± 2.41	57.71 ± 1.32	66.70 ± 1.03	78.70 ± 2.36
Artificial saliva	39.86 ± 1.04	49.74 ± 0.99	18.28 ± 0.87	48.80 ± 1.01	49.70 ± 1.04

**Table 4:** Disintegration behavior and pH values of extracts after 24 h of film disintegration in various media at 37.1 °C.

Formulation	Medium	pH	Observed Disintegration Behavior
FR1	Distilled water	7.10	Partially disintegrated
	Phosphate buffer	6.94	Partially disintegrated
	Glycol	8.04	Intact
	DMSO	11.83	Intact, rolled
	Artificial saliva	7.60	Partially disintegrated
FR2	Distilled water	7.33	Partially disintegrated
	Phosphate buffer	6.91	Intact, bulged
	Glycol	7.86	Intact
	DMSO	11.71	Intact, rolled
	Artificial saliva	7.36	Intact, flexible
FR3	Distilled water	7.20	Complete disintegration
	Phosphate buffer	6.90	Partially disintegrated
	Glycol	7.76	Intact
	DMSO	11.86	Intact
	Artificial saliva	7.65	Intact, flexible
FE1	Distilled water	7.12	Partially disintegrated
	Phosphate buffer	6.90	Partially disintegrated
	Glycol	7.98	Intact
	DMSO	11.38	Intact, rolled
	Artificial saliva	7.43	Partially disintegrated
FE2	Distilled water	7.43	Complete disintegration
	Phosphate buffer	6.91	Partially disintegrated
	Glycol	8.05	Intact
	DMSO	11.31	Intact, rolled
	Artificial saliva	7.37	Complete disintegration
FE3	Distilled water	6.80	Complete disintegration
	Phosphate buffer	6.88	Partially disintegrated
	Glycol	7.54	Intact
	DMSO	11.33	Intact, transparent
	Artificial saliva	7.42	Intact, flexible

**Table 5:** Composition of optimized polymer films.

Polymers and Additives	Formulation					
	FR1	FR2	FR3	FE1	FE2	FE3
PVA	+	+	+	+	+	+
PVP	+	+	-	+	+	+
MC 400 cP	+	-	+	+	-	+
NaCMC	+	+	+	+	+	-
MCA 15C	-	+	-	-	+	-
Water (W)	+	+	-	+	+	-
Propylene Glycol (PGE)	+	+	+	+	+	+
Ethanol 25% (EtOH) (aqueous solution)	+	+	+	+	+	+

**Table 6:** Quantitative composition of formulation FR1.

Ingredient	Quantity
PVA 2.5%	14.31 g
PVP 5%	8.45 g
RSV	0.395 mg in 3 mL 25% EtOH
MC 400 cP 5%	22.76 g
NaCMC 4%	19.51 g
Water	2 mL
Glycerol 85%	1.95 g

**Table 7:** Quantitative composition of formulation FE1.

Ingredient	Quantity
PVA 2.5%	14.31 g
PVP 5%	8.45 g
*R. japonica* extract	200 mg in 3 mL 25% EtOH
MC 400 cP 5%	22.76 g
NaCMC 4%	19.51 g
Water	2 mL
Glycerol 85%	1.95 g

**Table 8:** Quantitative composition of formulation FR2.

Ingredient	Quantity
PVA 2.5%	14.29 g
PVP 5%	8.45 g
RSV	0.395 mg in 3 mL 25% EtOH
MCA 15C%	22.75 g
NaCMC 4%	19.52 g
Water	2 mL
Glycerol 85%	1.95 g

**Table 9:** Quantitative composition of formulation FE2.

Ingredient	Quantity
PVA 2.5%	14.29 g
PVP 5%	8.45 g
*R. japonica* extract	200 mg in 3 mL 25% EtOH
MCA 15C 3%	22.75 g
NaCMC 4%	19.52 g
Water	2 mL
Glycerol 85%	1.95 g

**Table 10:** Quantitative composition of formulation FR3.

Ingredient	Quantity
PVA 2.5%	22.24 g
RSV	0.395 mg in 3 mL 25% EtOH
MC 400 cP 5%	33.35 g
NaCMC 4%	10 g
Glycerol 85%	2.4 g

**Table 11:** Quantitative composition of formulation FE3.

Ingredient	Quantity
PVA 2.5%	38 g
PVP 5%	10 g
*R. japonica* extract	200 mg in 3 mL 25% EtOH
MC 400 cP 5%	57 g
Glycerol 85%	5 g

**Table 12:** Optimized films production methodology.

FR1/FE1	FR2/FE2	FR3	FE3
PVA + PVP 🔽 Mixing 30 min Cooling (−18 °C) 18 h 🔽 + Extract/RSV, Mixing 2 h Cooling (−18 °C) 17 h 🔽 Ultrasounds 15 min Freezing 1 h Ultrasounds 15 min Cooling (−18 °C) 19 h Mixing 1.5 h 🔽 + MC 400 cP + NaCMC, Mixing 2 h, Cooling (−18 °C) 19 h 🔽 + Glycerol, Mixing 3 h, Cooling (−18 °C) 16 h 🔽 + Water, Mixing 1.5 h 🔽 5 × freezing and 30 min of mixing after each freezing 🔽 4 × sonification, 30 min after each cooling for 45 min in −18 °C 🔽 Pouring 30 g/vacuum–0.04 Pa 🔽 Drying 48 h at 37 °C	PVA + PVP 🔽 Mixing 30 min Cooling (−18 °C) 18 h 🔽 + Extract/RSV, Mixing 2 h, Cooling (−18 °C) 17 h 🔽 Ultrasounds 15 min Freezing 1 h Ultrasounds 15 min Cooling (−18 °C) 19 h Mixing 1.5 h 🔽 + MC A15C + NaCMC, Mixing 12 h, Cooling 19 h 🔽 + Glycerol, Mixing 3 h, Cooling (−18 °C) 16 h 🔽 + Water, Mixing 1.5 h 🔽 5 × freezing, 30 min of mixing after each freezing 🔽 4 × sonification, 30 min after each cooling for 45 min in −18 °C 🔽 Pouring 30 g/vacuum–0.04 Pa 🔽 Drying 48 h at 37 °C	PVA + RSV, Mixing 2 h Cooling (−18 °C) 17 h 🔽 Ultrasounds 15 min Freezing 1 h Ultrasounds 15 min Cooling (−18 °C) 19 h Mixing 1.5 h 🔽 + NaCMC, Mixing 2 h, Cooling (−18 °C) 19 h 🔽 + Glycerol, Mixing 3 h, Cooling (−18 °C) 16 h 🔽 5 × freezing + 30 min of mixing after each freezing 🔽 4 × sonification, 30 min after each cooling for 45 min in −18 °C 🔽 Pouring 30 g/vacuum–0.04 Pa 🔽 Drying 48 h at 37 °C	PVA + PVP 🔽 Mixing 30 min Cooling (−18 °C) 18 h 🔽 + Extract, Mixing 2 h, Cooling (−18 °C) 17 h 🔽 Ultrasounds 15 min Freezing 1 h Ultrasounds 15 min Cooling (−18 °C) 19 h Mixing 1.5 h 🔽 + MC 400 cP Mixing 2 h, Cooling (−18 °C) 19 h 🔽 + Glycerol, Mixing 3 h, Cooling (−18 °C) 16 h 🔽 5 × freezing and 30 min of mixing after each freezing 🔽 4 × sonification, 30 min after each cooling for 45 min in −18 °C 🔽 Pouring 30 g/vacuum–0.04 Pa 🔽 Drying 48 h at 37 °C

## Data Availability

The data presented in this study are available on request from the corresponding author.

## Abbreviations

API	Active pharmaceutical ingredient
DMSO	Dimethyl sulfoxide
EtOH	Ethanol
MC 400 cP	Methylcellulose, viscosity 400 cP
MCA 15C	Methylcellulose, viscosity 15 cP
NaCMC	Sodium carboxymethylcellulose
PGE	Propylene glycol
PVA	Polyvinyl alcohol
PVP	Polyvinylpyrrolidone
RSV	Resveratrol
W	Distilled water
